# Supplementary material for: Reticulocalbin 3 Is a Novel Mediator of Glioblastoma Progression
Source: Cancers (Basel). 2023 Mar 28;15(7):2008. doi: 10.3390/cancers15072008 (PMC10093618; doi:10.3390/cancers15072008)
Supplement: Supplementary file 1 [file cancers-15-02008-s001.zip › cancers-2236871-supplementary.pdf]

Supplementary Figure S1

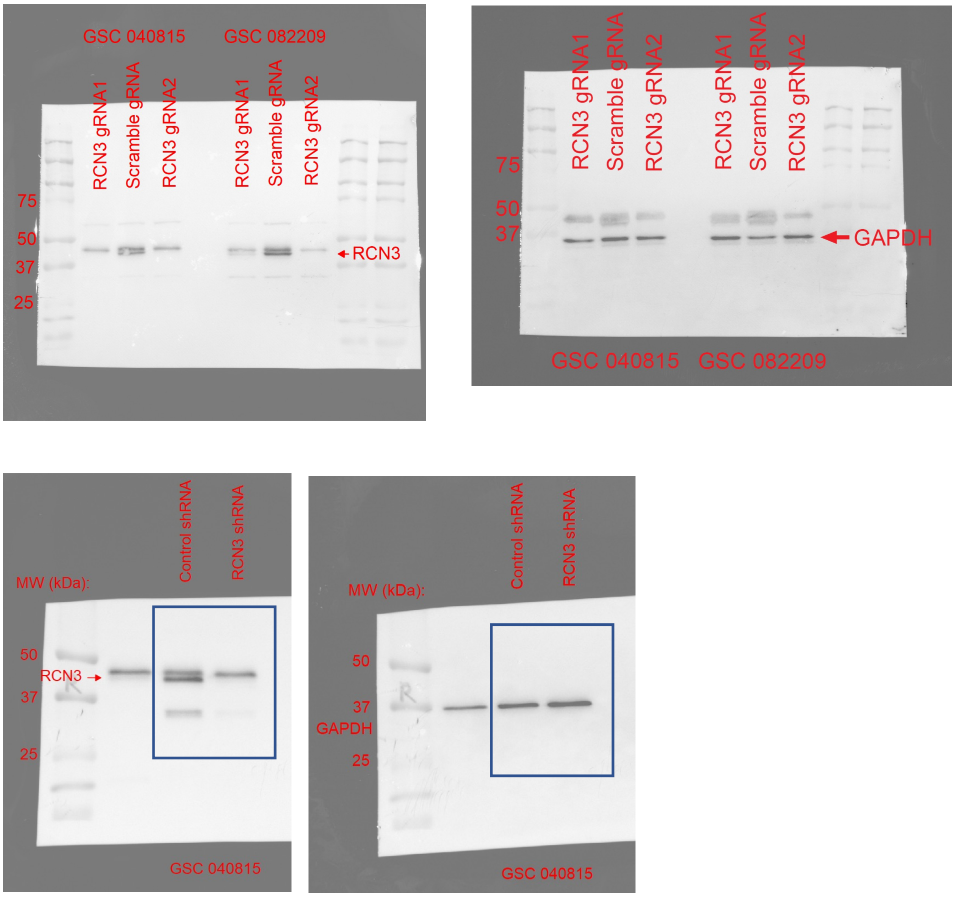

Supplementary Fig. S1: Uncropped western blots

Supplementary Figure S2

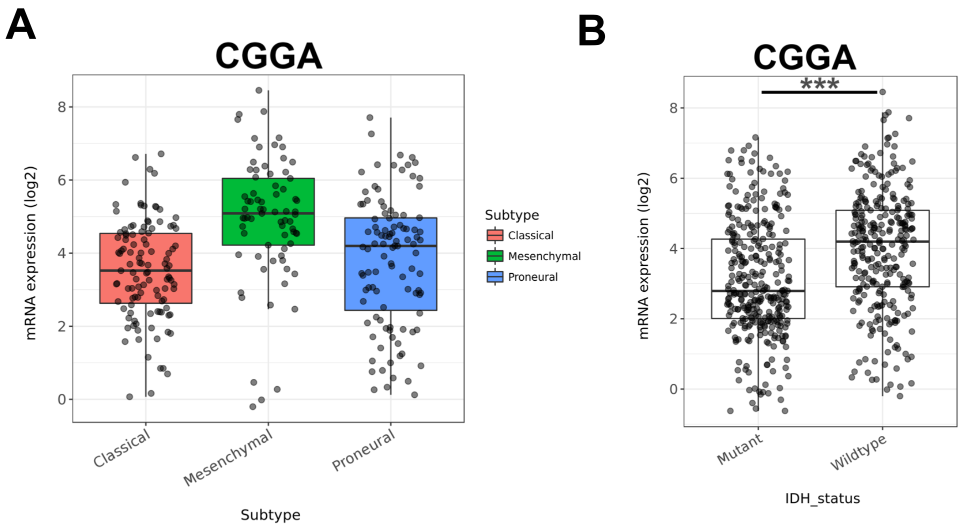

Supplementary Fig. S2: Expression of RCN3 in glioblastoma molecular subtypes (A) and IDH wild-type and mutant tumors (B) from CGGA patient data sets.

Supplementary Figure S3

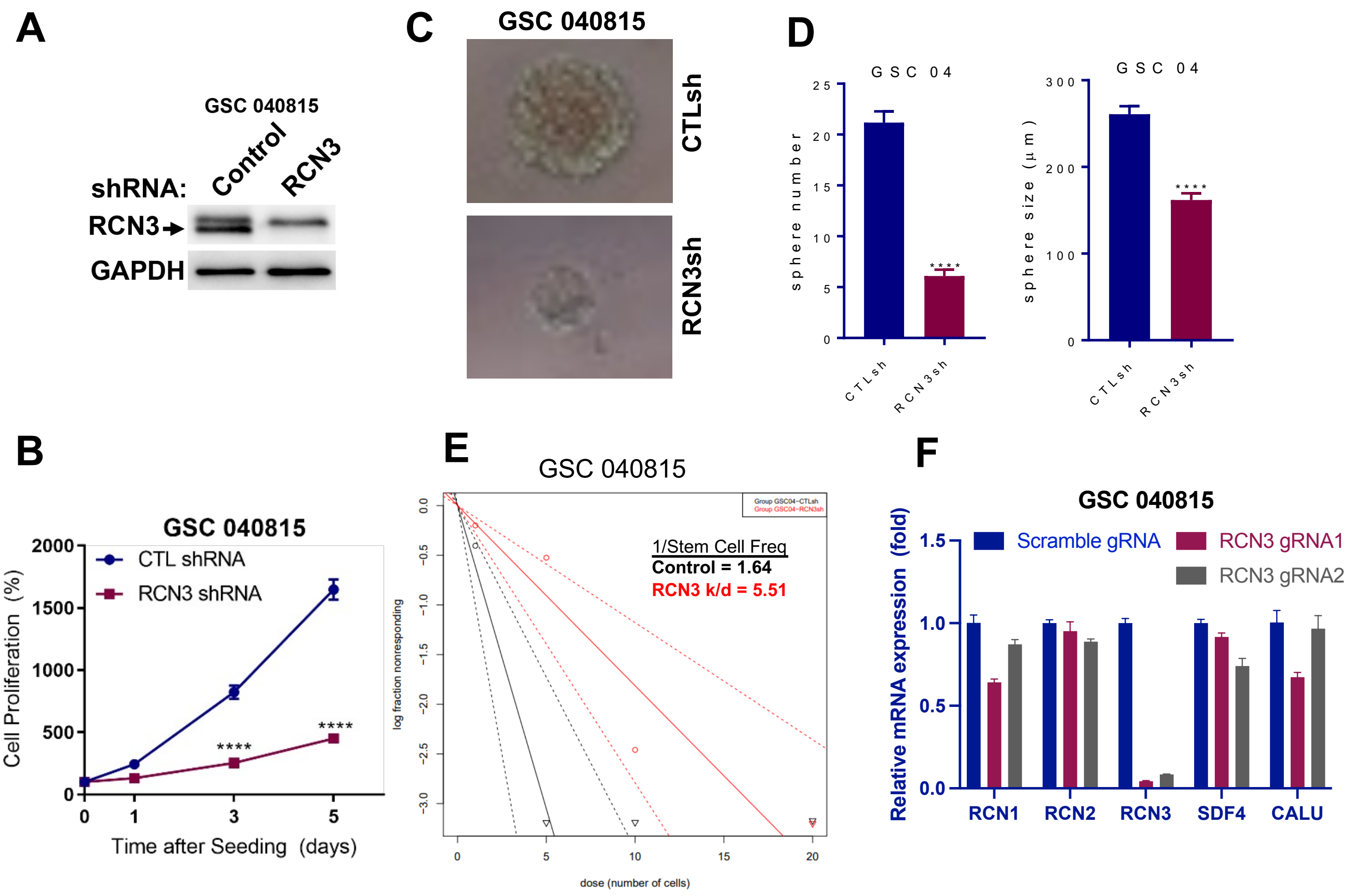

Supplementary Fig. S3: RCN3 knockdown reduced the proliferation and self-renewal of GSCs. (A) GSC040815 cells were transduced with either control or RCN3 shRNA, and knockdown of RCN3 was confirmed using western blotting. (B) Cell proliferation rates of control and RCN3 knockdown GSCs were determined using CellTiter-Glo assays. (C-D) Neurosphere formation of control and RCN3 knockdown GSCs was determined. (E) Self-renewal ability of control and RCN3 knockdown GSCs was determined by extreme limiting dilution assays and quantified as the reciprocal of stem cell frequency (1/Stem Cell Freq). The higher the self-renewal ability, the lower 1/Stem Cell Freq. (F) Expression of CREC family members in control and RCN3 knockout GSCs was determined using RT-qPCR assays. ****$p<0.0001$, by t-test or one-way ANOVA

Supplementary Figure S4

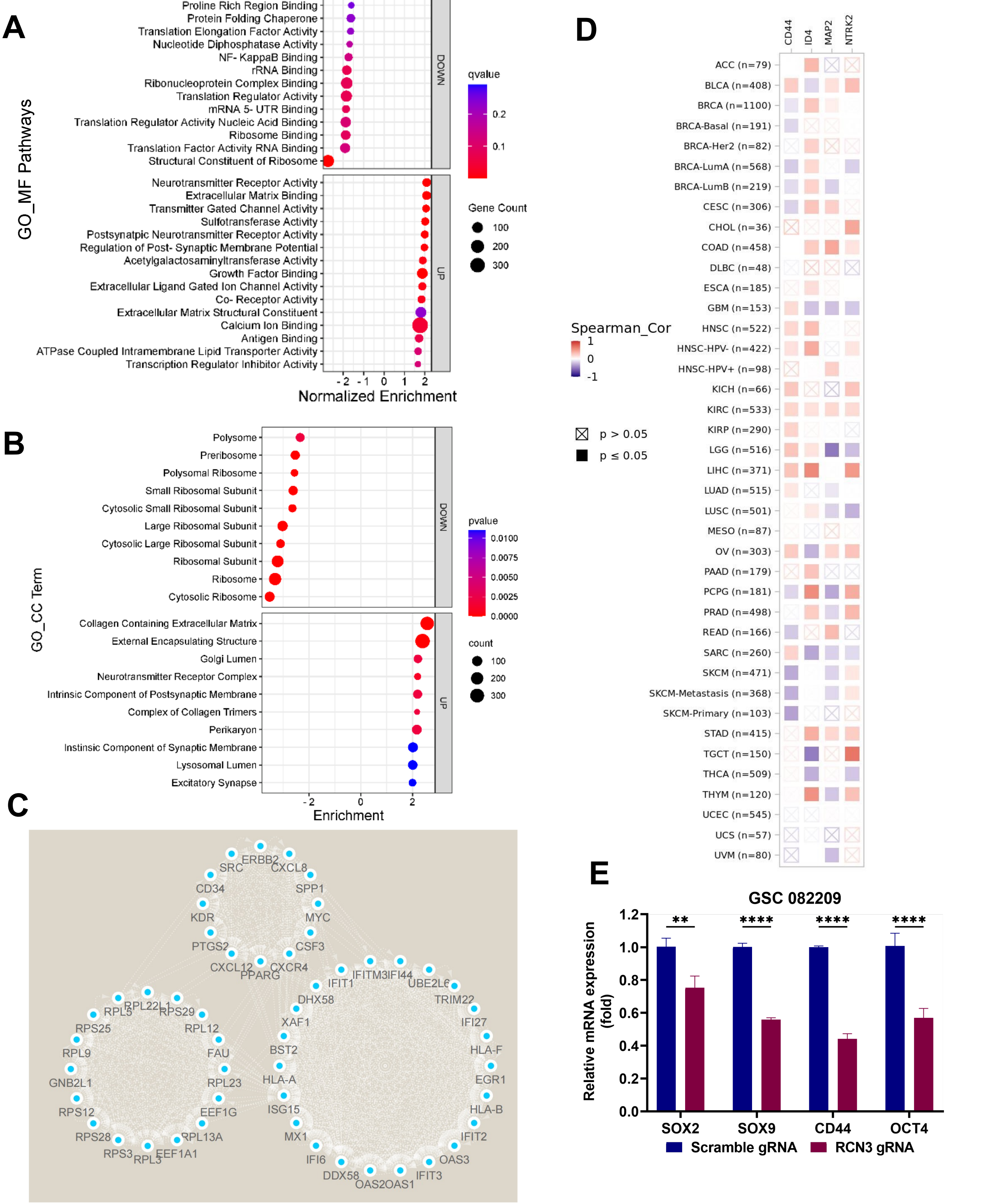

Supplementary Fig. S4: Top enriched GO terms of molecular functions (A), and cellular components (B) from GSEA analysis were shown in bubble plots. (C) Key protein-protein-interaction network of DEGs analyzed with the STRING platform and visualized with MCODE application in Cytoscape software. (D) Heatmap from TIMER2.0 database showing the expression correlations between RCN3 and stemness marker CD44 and differentiation markers in pan-cancers. (E), RT-qPCR confirming the downregulation of stemness genes in RCN3 knockdown GSCs. **p<0.01, ****p<0.001, by Student's t-test.

Table S1

Primer	Sequence 5'-3'
RCN3-F	TGTGGCGACCATCAGTTCTG
RCN3-R	GTCGTACTGGAAGTTCCCGTG
RCN1-F	CAGACCTCAATGGTGACCTGA
RCN1-R	CCGCAATATACTCATCCTGATCC
RCN2-F	TTCAGGTCCCGGTTTGAGTCT
RCN2-R	TCAAGCCTGCCATCGTTATCT
SDF4-F	TCCAAGGTGGATGTGAACACT
SDF4-R	GGCCTTTACTCGCCAAAAACT
CALU-F	AATAGACGCGGATAAAGATGGGT
CALU-R	GCCATTGGTTTTCAACATTGTCA
SOX9-F	AGCGAACGCACATCAAGAC
SOX9-R	CTGTAGGCGATCTGTTGGGG
OCT4-F	CTTGAATCCCGAATGGAAAGGG
OCT4-R	GTGTATATCCCAGGGTGATCCTC
SOX2-F	GCCGAGTGGAAACTTTTGTCG
SOX2-R	GGCAGCGTGTACTTATCCTTCT
CD44-F	CTGCCGCTTTGCAGGTGTA
CD44-R	CATTGTGGGCAAGGTGCTATT
RPS6-F	TGGACGATGAACGCAAACTTC
RPS6-R	TTCGGACCACATAACCCTTCC
RPL27-F	AATGCCCACAAGGTACTCTGT
RPL27-R	CTTGCGTTTAAGAGCAGGATCT
RPL8-F	AAGGGCATCGTCAAGGACATC
RPL8-R	CAGCTCCGTCCGCTTCTTAAA
RPS3-F	AGAGGAAGTTTGTCGCTGATG
RPS3-R	GCACCTCAACTCCAGAGTAGC
RPL3-F	TGAAGAGCTTCCCTAAGGATGA
RPL3-R	CTTCCCGCACGATGTGAGTC
RPS8-F	GCTCAGAGTGTTGTACTCGTAAA
RPS8-R	AGCACGATGCAATTCTTCACC
RPL11-F	AAAGGTGCGGGAGTATGAGTT
RPL11-R	TCCAGGCCGTAGATACCAATG
RPL23-F	TCCTCTGGTGCGAAATTCCG
RPL23-R	CGTCCCTTGATCCCCTTCAC
RPS14-F	CCATGTCACTGATCTTTCTGGC
RPS14-R	TCATCTCGGTCTGCCTTTACC
HSPA5-F	CATCACGCCGTCCTATGTCG
HSPA5-R	CGTCAAAGACCGTGTTCTCG
ATF4-F	CTCCGGGACAGATTGGATGTT
ATF4-R	GGCTGCTTATTAGTCTCCTGGAC
XBP1-F	CCCTCCAGAACATCTCCCCAT
XBP1-R	ACATGACTGGGTCCAAGTTGT
TBP-F	TTGGGTTTTCCAGCTAAGTTCT
TBP-R	CCAGGAAATAACTCTGGCTCA
GAPDH-F	TCGACAGTCAGCCGCATCT
GAPDH-R	CTAGCCTCCCGGGTTTCTCT
